# Supplementary material for: Efficacy and Tolerance of Antipsychotics Used for the Treatment of Patients Newly Diagnosed with Schizophrenia: A Systematic Review and Meta-Analysis
Source: Pharmacy (Basel). 2023 Nov 10;11(6):175. doi: 10.3390/pharmacy11060175 (PMC10661248; doi:10.3390/pharmacy11060175)
Supplement: Supplementary file 1 [file pharmacy-11-00175-s001.zip › pharmacy-2642602-supplementary.pdf]

Title: **Efficacy and tolerance of antipsychotics used for in the treatment of patients newly diagnosed with schizophrenia: A Systematic review and meta-analysis**

Supplementary 1: Selected studies summary (direct quotations)

Citation and country	Design, site, registration, ethics and consent	Age and randomised sample	Objectives of study and Definition of drug naivety	Interventions (dose and number of patient) and concomitant medication use with psychotropic medications	Wash-out follow-up Period	Funding reported
Amr *et al.* (2013) Jordan Multi-centre [30]	Double blinded Randomised controlled trial Hospitals Registered Approved Consent obtained	18–60 years n=73	Evaluates the efficacy and tolerability of quetiapine versus haloperidol for first-episode schizophrenia in the outpatient setting. Patients with current or past use of APDs for any treatment of psychosis is excluded.	Quetiapine (200 mg/d) or haloperidol (5 m/d) Concomitant medication use: lorazepam and zopiclone. lorazepam (1–4 mg/d) / zopiclone (3.75–7.5 mg/d) anticholinergic biperiden (2–8 mg/d)	NR 12 weeks Oct 2009 to Sep 2011	NR
Crespo-Facorro *et al.* (2013) Spain Single centre [39]	Prospective, randomised, flexible dose, open-label study University hospital Registered Approved Consent obtained	15–60 years n=202	To compare the clinical effectiveness of aripiprazole, ziprasidone and quetiapine in the treatment of first-episode schizophrenia spectrum disorders at 1 year. Patients have not received previous APD treatment regardless of the duration of psychosis.	5–30 mg/day of aripiprazole (n=78), 40-160 mg/day of ziprasidone (n=62), or 100–600 mg/day of quetiapine (n=62) Concomitant medication use: antimuscarinic medication, lormetazepam and clonazepam. antidepressants and mood stabilizers	2–4-day 12 months Oct 2005 to Jan 2011	Yes
Crespo-Facorro *et al.* (2014) Spain Single centre [38]	Prospective, randomised, flexible-dose, open-label study University hospital Registered Approved Consent obtained	15–60 years n=202	To compare the clinical effectiveness in the short-term of aripiprazole, ziprasidone and quetiapine in the treatment of first-episode non-affective psychosis individuals. No prior treatment with APD or, if previously treated, a total lifetime of adequate APD treatment of less than 6 weeks.	Aripiprazole 5–30 mg/day (n=78), Ziprasidone40–160 mg/day (n= 62), or Quetiapine 100–600 mg/day (n=62) Concomitant medication use: lormetazepam, clonazepam, sertraline, lithium	2–4-day 3 months Oct 2005 to Jan 2011	Yes
Crespo-Facorro *et al.* (2017) Spain Single centre [40]	Randomised, flexible-dose, open-label, longitudinal study Registered Approved Consent obtained	15-60 years n=141	To explore the differential effect of three widely used prolactin-sparing APDs, aripiprazole, quetiapine and ziprasidone, on prolactin plasma levels in first episode non-affective psychosis during a 1 year of treatment. No previous treatment with APDs or, if formerly treated, a lifetime of adequate APD treatment of less than 6 weeks.	Aripiprazole (n=56), 5–30 mg/day quetiapine (n=36) 100-600 mg/day ziprasidone (n=49) 40–160 mg/day Concomitant medication use: antimuscarinic agents, antidepressants, mood stabilizers, hypnotics, were permitted if clinically needed.	NR 1 year October 2005 to January 2011	NR
Girgis *et al.* (2011) China Single centre [37]	Double-blind randomised Mental health centre NR Approved Consent obtained	15-42 years n=164	NR No previous APD treatment or a maximum of 14 days of prior use.	Clozapine (n=80) or chlorpromazine (n=80) NR	NR 9 years NR	Yes
Gómez-Revuelta *et al.* (2018) Spain	Prospective, randomised, flexible dose, open-label study University hospital	15-60 years n=202	To compare the clinical effectiveness of aripiprazole, ziprasidone, and quetiapine in the treatment of first episode of psychosis at 3-year follow-up.	Quetiapine 100 to 600 mg/d (133.33–800 mg/d of chlorpromazine), ziprasidone 40 to 160 mg/d (66.67–266.67 mg/d of chlorpromazine), and aripiprazole 5 to 30 mg/d (66.67–400 mg/d of	2-4-day 3 years October 2005 - January 2011	NR

Citation and country	Design, site, registration, ethics and consent	Age and randomised sample	Objectives of study and Definition of drug naivety	Interventions (dose and number of patient) and concomitant medication use with psychotropic medications	Wash-out follow-up Period	Funding reported
Single centre [42]	Registered<br>Approved<br>Consent obtained		No prior treatment with APD medication or, if previously treated, a total lifetime of adequate APD treatment of less than 6 weeks.	chlorpromazine)<br>Concomitant medication use: Anticholinergic medications, lormetazepam and clonazepam, were permitted for clinical reasons. No anticholinergic agents were administered prophylactically. Antidepressants and mood stabilizers were permitted if clinically needed		
Gómez-Revuelta *et al.* (2020) Spain Single centre [35]	Prospective, randomised, flexible-dose, open-label clinical trials<br>University hospital<br>NR<br>Approved<br>Consent obtained	15-60 years<br><br>n=376	To compare the clinical effectiveness of olanzapine, risperidone, haloperidol, aripiprazole, ziprasidone, and quetiapine in the treatment of first episode of psychosis at 3-year follow-up.<br>No prior treatment with APD or, if previously treated, a total lifetime of adequate APD treatment of less than 6 weeks.	Olanzapine: 5–20 mg/d (100–400 CPZeq) n: 55, Risperidone3–6 mg/d (150– 300 CPZeq) n:63, Haloperidol 3–9 mg/d (150–450 CPZeq) n:56, Aripiprazole 5–30 mg/d (66,67–400 PZeq) n:78, Ziprasidone 40–160 mg/d (66.67– 266.67 CPZeq) n:62, Quetiapine 100–600 mg/d (133.33–800 CPZeq) n:62<br>Concomitant medication use: Anticholinergic medication, lormetazepam, and clonazepam. No anticholinergic agents were administered prophylactically. Antidepressants and mood stabilizers were permitted if clinically needed	2-4-day<br>3 years<br>2001–2004 and 2005–2011	NR
Gómez-Revuelta *et al.* (2021) Spain Single centre [31]	Prospective, randomised, flexible-dose, open-label clinical Trial<br>University Hospital<br>Registered<br>Approved<br>Consent obtained	15-60 years<br>n=266	To compare the effectiveness of aripiprazole and risperidone in<br>the treatment of the acute phase after a first episode psychosis.<br>No prior treatment with APD (naïve) or, if previously treated, a total lifetime of adequate APD treatment of less than 6 weeks.	Risperidone (n=130). 3–6 mg/d (300–600 CPZeq) and Aripiprazole (n=136) 5–30 mg/d (100–600 CPZeq).<br>Concomitant medication use: Anticholinergic medication, lormetazepam, and clonazepam were permitted for clinical reasons. No anticholinergic agents were administered prophylactically. Antidepressants and mood stabilizers were permitted if clinically needed.	2-4-day<br>6-weeks<br>February 2011 -October 2018	Yes
Green *et al.* (2006) North America and Western Europe Multi-centre [34]	Double-blind randomised<br>NR<br>Registered<br>Approved<br>Consent obtained	16-40 years<br>n=260	To examine the effectiveness of the SGAs olanzapine and the FGAs haloperidol in patients experiencing their first episode of a schizophrenia-related psychotic disorder over a 2-year.<br>Previous treatment with APD medications was allowed, but for no more than 16 cumulative weeks, patients with a previous trial of clozapine or those treated with an injectable depot neuroleptic within 3 months of study entry were excluded.	During the acute treatment phase,<br>initial dose titration ranges for first 6 weeks were olanzapine 5–10 mg/day and haloperidol 2–6 mg/day; for the second 6 weeks of the acute phase and for entire<br>continuation phase, the allowed doses were olanzapine 5- 20 mg/day and haloperidol 2 to 20 mg/day.<br>Concomitant medication use: Chloral hydrate (500 to 2000 mg/day), lorazepam (1 to 8 mg/day) or diazepam (5 to 40 mg/day), anticholinergics (benztropine or biperiden up to 6	2-14-day<br>2 years<br>NR.	Yes

Citation and country	Design, site, registration, ethics and consent	Age and randomised sample	Objectives of study and Definition of drug naivety	Interventions (dose and number of patient) and concomitant medication use with psychotropic medications	Wash-out follow-up Period	Funding reported
				mg/day), propranolol (10 to 80 mg/day) or procyclidine (oral or intramuscular administration of 5 to 10 mg, two to three times daily, for a maximum dose of 30 mg/day)/ fluoxetine, lithium or valproate.		
Grootens *et al.* (2009)<br>Netherlands and Belgium<br>NR [25]	Double-blind, parallel groups, randomised<br>Hospital<br>NR<br>NR<br>NR	18–40 years<br>n=74	The aim of the present study was to compare the efficacy and tolerability of ziprasidone and olanzapine in patients with recent-onset schizophrenia.<br>Maximum lifetime APD treatment <16 weeks participated in the study.	Olanzapine (n = 35) 14 mg, ziprasidone (n = 39), 104 mg,<br>Concomitant medication use: biperiden, propranolol, benzodiazepines, lithium, antidepressants.	NR<br>8 weeks<br>NR	NR
Kahn *et al.* (2008)<br>13 European countries and Israel<br>Multi-centre [36]	Double-blind randomised<br>Psychiatric Hospital<br>Registered<br>Approved<br>Consent obtained	18-40 years<br>n=498	This study examined effectiveness of several second generation APDs (SGA) in first episode schizophrenia with minimal prior exposure to APD treatment.<br>Minimal prior exposure to APD treatment over a one year period.	Haloperidol= 103, amisulpride = 104, olanzapine = 105, quetiapine= 104, ziprasidone = 82<br>Concomitant medication use: antidepressants, anticholinergics.	NR<br>NR<br>December 2003 - October 2004	Yes
Lieberman *et al.* (2003), China<br>Single centre [33]	Double-blind randomised flexible dose<br>NR<br>NR<br>Approved<br>Consent obtained	15-42 years<br>n=164	To examine efficacy and safety of chlorpromazine (CPZ) and clozapine (CLZ) in treatment naive patients experiencing their first episode of schizophrenia.<br>No prior treatment with APD or, if previously treated, a total lifetime usage of less than 14 days.	Mean dosage of 400 and 300, respectively, for CLZ, and 600 and 400 for CPZ.<br>NR	NR<br>52 weeks<br>October 1995 - December 1998	Yes
Lieberman *et al.* (2003)<br>North America and Western Europe<br>Multi-centre [28]	Double-blind randomised.<br>NR<br>NR<br>Approved<br>Consent obtained	16–40 years<br>NR	This study compared the acute and long-term effectiveness of haloperidol with that of olanzapine in patients with first-episode psychosis in a large, controlled clinical trial.<br>Patients were excluded if had previously received APD treatment for more than 16 cumulative weeks, had been treated with clozapine at anytime in their lifetime, or had been treated with an injectable depot neuroleptic within less than three dosing intervals before study entry.	5–10 mg/day for olanzapine and 2–6 mg/day for haloperidol.<br>Concomitant medication use: 500–2000 mg/day of chloral hydrate, 1–8 mg/day of lorazepam, and 5–40 mg/day of diazepam.	2-14-day<br>NR<br>NR	Yes
McEvoy *et al.* (2007)	Randomised, double-blind, flexible dose	16–40 years<br>NR	Evaluated the overall effectiveness (as measured by treatment discontinuation rates) of	Olanzapine (2.5–20 mg/day), quetiapine (100–800 mg/day), or risperidone (0.5–4 mg/day).	NR<br>52 weeks	Yes

Citation and country	Design, site, registration, ethics and consent	Age and randomised sample	Objectives of study and Definition of drug naivety	Interventions (dose and number of patient) and concomitant medication use with psychotropic medications	Wash-out follow-up Period	Funding reported
USA<br>Multi-centre [29]	Hospital<br>Registered<br>Approved<br>Consent obtained		olanzapine, quetiapine, and risperidone in patients early in the course of psychotic illness.<br>If a prior psychotic episode had remitted for 3 months or more or if they had prior APD treatment for more than 16 cumulative weeks.	Concomitant medication use: anticholinergic medications for acute EPS were permitted for up to a total of 2 weeks over the course of the trial. clinicians were encouraged to lower the dose of APD to relieve EPS. otherwise, adjunctive medications (prescribed to address an aspect of psychopathology inadequately controlled by the assigned APD) and concomitant medications (prescribed to treat a side effect or a comorbid medical illness) could be used without restriction.	NR	
Perkins *et al.* (2006)<br>USA<br>NR [44]	Double-blind randomised<br>NR<br>NR<br>Approved<br>Consent obtained	16–40 years<br>n=254	To assess efficacy and safety of olanzapine and haloperidol in patients experiencing a first episode of DSM IV schizophrenia, schizophreniform, or schizoaffective disorder. To examine the relationship between APD medication non-adherence and patient beliefs about: need for treatment, APD medication benefits, and negative aspects of APD treatment. To examine the relationship between medication non-adherence and treatment with either haloperidol or olanzapine, and objective measures of symptom response and side effects.<br>Patients were excluded if they had prior APD treatment more than 16 cumulative weeks.	Olanzapine (5–20 mg/day) or haloperidol (2–20 mg/day).<br>NR	NR<br>2 years<br>NR	Yes
Robinson *et al.* (2015)<br>Canada.<br>Multi-centre [32]	Double-blind randomised<br>Not-for-profit institutions<br>Registered<br>Approved<br>Consent obtained	15-40 years<br>=209	The outcomes of this study were to analyses of the longitudinal patterns of both positive and negative symptoms. Moreover, comparing metabolic effects and motor effects such as parkinsonism and akathisia.<br>NR	Aripiprazole (5–30mg/d) or risperidone (1–6mg/d).<br>Concomitant medication use: psychiatric medications were: benztropine for extrapyramidal symptoms (EPS); lorazepam or propranolol for akathisia; lorazepam (followed by sodium amytal or chloral hydrate as alternatives) for agitation or anxiety; lorazepam, zolpidem, or ramelteon for insomnia.	NR<br>12 weeks<br>December 2005 - April 2013	Yes
San *et al.* (2012)<br>Spain<br>Multi-centre [26]	Open-label flexible doses<br>Hospital<br>NR<br>Approved	Over 18 years<br><br>n=114	To compare the 12-month effectiveness of several second-generation APDs, with that of haloperidol in never-treated patients with first-episode psychosis.<br>Treatment naïve to APD.	Haloperidol (n=21), 1.5–8.5, olanzapine (n=25), 7.5–40, risperidone (n=20), 1.5–7.0, quetiapine (n=23) 100–1500 and ziprasidone (n=25).<br>Concomitant medication use: 40–240 mg/day	NR<br>52 weeks<br>January 2004 - December 2007	Yes

Citation and country	Design, site, registration, ethics and consent	Age and randomised sample	Objectives of study and Definition of drug naivety	Interventions (dose and number of patient) and concomitant medication use with psychotropic medications	Wash-out follow-up Period	Funding reported
	Consent obtained			benzodiazepines for the treatment of with the indication of anxiety, agitation or insomnia were permitted. the use of anticholinergics (biperiden) was allowed for clinically significant extrapyramidal signs, but not prophylactically. clinician's choice mood stabilizer and antidepressant prescription was documented at each visit.		
Schennach -Wolff *et al.* (2011)<br>Germany<br>Multi-centre [53]	Double-blind randomised<br>NR<br>NR<br>Approved<br>Consent obtained	Mean 30.64 years (± 9.95<br>n=224	To examine early response while concurrently evaluating the predictive validity of other baseline and early-course clinical as well as sociodemographic variables<br>Mean duration of untreated psychosis was 2.87 months (± 2.10).	Mean dosage of 4.17 mg (± 1.55) risperidone, mean dosage of 4.14 mg (± 1.70) haloperidol<br>Concomitant medication use: short-acting benzodiazepines lorazepam/biperiden (up to 6 mg/day) and for akathisia the betablocker propranolol (up to 80mg/day)	4–7 day<br>8-week<br>November 2000 - May 2004	Yes
Stauffer *et al.* (2011)<br>NR<br>Multi-centre [43]	Double-blind randomised<br>Hospital<br>NR<br>Approved<br>Consent obtained	16-40 years<br>n=225	To assess early response to APD therapy as a clinical marker of subsequent response in the treatment of patients with first-episode psychosis.<br>Prior APD treatment of more than 16 cumulative weeks in the patient's lifetime.	Olanzapine (5 to 20 mg/day) or haloperidol (2 to 20 mg/day).<br><u>Concomitant medication use:</u> chloralhydrate (500 to 2000 mg/day), lorazepam (1 to 8 mg/day), or diazepam (5 to40 mg/day)/ (benztropine mesylate or biperiden up to 6 mg/day), propranolol (10 to 80 mg/day), or procyclidine (oral or intramuscular administration up to 30 mg/day).	NR<br>12-week<br>NR	NR
Wang *et al.,* (2017)<br>China<br>Single centre [27]	Open label randomised controlled trial<br>Hospital<br>Registered<br>Approved<br>Consent obtained		To assess whether there are any differences in efficacy, acceptability, and safety between the five SGAs in patients with first episode schizophrenia.<br>NR	The mean (SD) dose of aripiprazole at study endpoint was 25.65 (6.45) mg/day; risperidone was titrated from the initial 0.5–1 mg up to a maximum of 6 mg a day, and the mean (SD) daily dose at study endpoint was 5 (2.51) mg; quetiapine was titrated from the initial 50 mg up to a maximum dose of 900 mg a day, and the mean (SD) dose of quetiapine at study endpoint was 634.52 (207.59) mg/day; olanzapine was titrated from the initial 5 mg up to a maximum dose of 20 mg a day, and its mean (SD) dose at study endpoint was 17.62 (3.4) mg/ day; and ziprasidone was titrated from the initial 40 mg up to a maximum dose of 160 mg a day, and its mean (SD) daily dose at study endpoint was 102.86 (49.57) mg.<br><u>Concomitant medication use:</u> benzodiazepines,	NR<br>NR<br>October 2012 - November 2014.	Yes

Citation and country	Design, site, registration, ethics and consent	Age and randomised sample	Objectives of study and Definition of drug naivety	Interventions (dose and number of patient) and concomitant medication use with psychotropic medications	Wash-out follow-up Period	Funding reported
				anticholinergics, or beta-blockers should be considered for the management of the emerged side effects. Haloperidol injection 5–10 mg a day was sometimes used for a short period of time when necessary (e.g., the patient with aggression and/or more violent behavioural disorders, such as agitation or hostility).		
Zipursky *et al.* (2005)<br>North America and Europe<br>Multi-centre [41]	Randomised double-blind clinical trial.<br>University hospital<br>NR<br>Approved<br>Consent obtained	16-40 years<br>n=263	To evaluate the extent, time course and predictors of weight gain and its effect on study retention among people with first-episode psychosis treated with olanzapine or haloperidol.<br>Treatment naïve OR total days of APD use <62.8.	Olanzapine or haloperidol<br><u>Concomitant medication use:</u> antidepressants and mood stabilisers could not be used during the first 12 weeks of the study. Following the 12-week acute treatment period, fluoxetine 10–60 mg/day could be prescribed for individuals meeting DSM–IV criteria for major depressive disorder. Lithium carbonate or valproate could be added if they failed to respond to fluoxetine or if they developed mania or a mixed affective sntate.	NR<br>12 weeks<br>March 1997 - July 2001	Yes

Supplementary 2: Selected studies additional information (direct quotations)

Citation	Gender Race	Diagnosis	Patient education	Parents education	Urban living	Living	Employment	Family psychiatric history	Inpatient	Drugs and alcohol
Amr *et al.* (2013) [30]	F=27<br>M=46	Paranoid or non-paranoid schizophrenia	SE=54	NR	NR	NR<br>n=42	N/R<br>n=60	NR	NR	NR
Crespo-Facorro *et al.* (2013) [39]	F=94<br>M=108<br>White=192<br>Others=10	Schizophrenia, brief psychotic disorder, unspecified psychotic disorder, schizophreniform disorder, schizoaffective disorder	PE=95	NE=92	n=150	n=93<br>n=135	n=39<br>n=90	n=48	n=134	T=119<br>C=79<br>A=108<br>O=39
Crespo-Facorro *et al.* (2014) [38]	NR<br>White=192	Schizophrenia, brief psychotic disorder, unspecified psychotic disorder, schizophreniform disorder, schizoaffective disorder	PE=95	NE=92	n=149	n=93<br>n=135	n=39<br>n=90	n=48	n=134	T=119<br>C=79<br>A=108<br>O=39
Crespo-Facorro *et al.* (2017) [40]	White=138	DSM-IV criteria for schizophrenia, schizophreniform disorder, brief psychotic disorder or schizoaffective disorder	PE=66	NE=68	n=99	n=100<br>NR	n=27<br>NR	n=35	n=91	T=80<br>C=48<br>A=73<br>O=N/R
Girgis *et al.* (2011) [37]	NR	schizophrenia or schizophreniform disorder, paranoid schizophrenia	NR	NR	NR	NR	NR	NR	NR	NR
Gómez-Revuelta *et al.* (2018) [42]	Male=53.5%<br>White=95%	Schizophrenia diagnosis	PE 100%	NE 45.8%	74.6%	46.3%<br>67.2%	19.4%<br>44.8%	23.8%	66.3%	T=58.9%<br>C=39.1%<br>A=53.5%<br>O=19.4%
Gómez-Revuelta *et al.* (2020) [35]	F=160<br>M=216<br>Caucasian=76	Schizophrenia diagnosis	PE=185	NE=196	n=281	n=203<br>n=281	n=74<br>n=169	n=83	n=133	T=220<br>C=161<br>A=204<br>O=86
Gómez-Revuelta *et al.* (2021) [31]	M=145<br>F=121<br>Caucasian=228	Schizophrenia, brief psychotic disorder, psychotic disorder, schizophreniform disorder, schizoaffective disorder	PE=126	NE=147	n=185	n=128<br>n=180	n=60<br>n=104	n=77	n=207	T=123<br>C=107<br>A=96<br>O=41
Perkins *et al.* (2006) [44]	M=208<br>F=46<br>Caucasian= 133<br>African=96<br>Other=25	Schizophrenia, schizophreniform disorder, schizoaffective disorder	NR	NR	NR	NR	NR	NR	NR	NR
Green *et al.* (2006) [34]	F=27<br>M=104<br>African=49<br>Asian=4<br>Caucasian=67	Schizophrenia, Schizophreniform disorder, schizoaffective disorder	NR	NR	NR	NR	NR	NR	NR	NR

Citation	Gender Race	Diagnosis	Patient education	Parents education	Urban living	Living	Employment	Family psychiatric history	Inpatient	Drugs and alcohol
	Hispanic=8<br>Other=3									
Grootens *et al.* (2009) [25]	F=13<br>M=61<br>NR	Schizophreniform disorder, schizophrenia, paranoid type, schizophrenia, disorganized type, schizophrenia, residual type, schizophrenia, undifferentiated type, schizoaffective disorder	NR	NR	NR	NR	NR	NR	NR	NR
Kahn *et al.* (2008) [36]	F=200<br>M=298<br>Caucasian= 94%	Schizophreniform disorder, schizoaffective disorder, schizophrenia	NR	NR	NR	87%<br>NR	NR<br>53%	NR	NR	T=N/R<br>C=N/R<br>A=10%<br>O=17%
Lieberman *et al.* (2003) [33]	F=79<br>M=85<br>Chinese=100%	Schizophrenia	NR	NR	NR	NR	NR	NR	NR	NR
Lieberman *et al.* (2003) [28]	F= 48<br>M=215<br>African=99<br>Caucasian=139<br>East Asian=9<br>Hispanic=12<br>Other=4	Schizophrenia, Schizoaffective disorder, Schizophreniform disorder	NR	NR	NR	NR	NR	NR	NR	NR
McEvoy *et al.* (2007) [29]	F=108<br>M=292<br>White=205<br>Black=172<br>Other=23	Schizophrenia, Schizophreniform disorder, schizoaffective disorder	NR	NR	NR	NR	NR	NR	NR	NR
Robinson *et al.* (2015) [32]	F=58<br>M=140<br>African=37%<br>Asian=20%<br>Caucasian=24%<br>Hispanic=10%<br>Other=9%	Schizophrenia, Schizophreniform disorder,<br>Schizoaffective disorder,<br>Psychotic disorder	NR	NR	NR	NR<br>n=5	NR	NR	NR	NR
San *et al.* (2012) [26]	M=85<br>F=29<br>NR	Schizophrenia, brief psychotic disorder, psychotic disorder, schizophreniform disorder, schizoaffective disorder, bipolar disorder, substance induced psychosis	PE=53<br>SE=43<br>UE=18	NR	NR	NR<br>n=94	NR	NR	NR	T=75<br>C=64<br>A=87<br>O=24
Schennach	F=94	Schizophrenia	NR	NR	NR	NR	NR	NR	NR	NR

Citation	Gender Race	Diagnosis	Patient education	Parents education	Urban living	Living	Employment	Family psychiatric history	Inpatient	Drugs and alcohol
-Wolff *et al.* (2011) [53]	M=130<br>NR									
Stauffer *et al.* (2011) [43]	F=39<br>M=186<br>African= 86<br>Caucasian=115	Schizophrenia, Schizophreniform disorder, schizoaffective disorder	N/R	NR	NR	NR	NR	NR	NR	NR
Wang *et al.* (2017) [27]	F=81<br>M=94<br>NR	First-episode drug-naïve, schizophrenia	NR	NR	NR	NR	NR	NR	NR	NR
Zipursky *et al.* (2005) [41]	M=214<br>F=21<br>White=139, 53%	Schizophrenia, Schizophreniform, Schizoaffective	NR	NR	NR	NR	NR	NR	NR	NR

Patient education: No education (NE), Primary education (PE), Secondary education (SE), University education (UE)
Parents education: educated (E), Not educated (NE)
Parents education: educated (E), Not educated (NE)
Living: Not living alone (NLA), Living alone (LA)
Student (S) or Unemployed (UEM), Employed (EM)
Tobacco (T), Cannabis (C), Alcohol (A), Other drugs (O)
NR: Not Reported

Supplementary 3: Selected studies limitations

Citation	Study reported limitations	Reviewer Comments
Amr *et al.* (2013) [30]	The sample size in this study was relatively small and the study design took place in two-centres which are both considered as the limitation of this study.	This study will be used in the categories of short term treatment study. It will be included in analysis of clinical efficacy, extrapyramidal and other side effect, concomitant medications and discontinuation rate and reasons.
Crespo-Facorro *et al.* (2013) [39]	The fact that patients and observers knew the medications prescribed causes limitation to this study as it may have involuntarily biased the outcomes. grants from AstraZeneca, Pfizer, Bristol-Myers Squibb and Johnson & Johnson provided but No pharma industry has participated in any stages of the study.	This study will be used in the categories of long term treatment study. It will be included in analysis of clinical efficacy , extrapyramidal and other side effect, concomitant medications and discontinuation rate and reasons for aripiprazole, ziprasidone and quetiapine.
Crespo-Facorro *et al.* (2014) [38]	This study was open label study .	This study will be used in the categories of short term treatment study. It will be included in analysis extrapyramidal and other side effect, and discontinuation rate and reasons for aripiprazole, ziprasidone and quetiapine. Part of this research data including clinical efficacy and concomitant medication use was missing from its report. I have contacted the author but unfortunately, they did not get back to me
Crespo-Facorro *et al.* (2017) [40]	It was an open trial . high numbers of patients switched their initial assigned APDs during follow-up. Also, the results of this study could be biased since women showed more HPRL than men, since cut-off levels for HPRL but are not influenced by gender, despite the fact that women have greater prolactin levels than men. In addition, risk factors such as antidepressant or oral contraception use or thyroid dysfunction, or sexual dysfunction, which could influence prolactin level. Also, personal sexual problems were not measured too.	This study will be used in the categories of long term treatment study. It will be included in analysis extrapyramidal and other side effect, and discontinuation rate and reasons for aripiprazole, ziprasidone and quetiapine. Lastly, some information such as clinical efficacy and detail percentage of concomitant medications were not present despite contacting the author.
Girgis *et al.* (2011) [37]	initially patients were receiving randomised, double-blind treatment , however, an open, naturalistic treatment was conducted for additional 7 years, therefore, a notable crossover between the two groups occurred. In addition, funds and medications from the Novartis Pharmaceutical Company received .	This study will be used in the categories of long term treatment study. It will be included in analysis of the side effect, and discontinuation reasons for clozapine and chlorpromazine.
Gómez-Revuelta *et al.* (2018) [42]	One of the limitations in this study that could have involuntarily biased the outcomes are that patients and observers were not blinded to treatments. Treatment compliance was either self-reported or by close observers and not from APD blood levels which could have affected the accuracy of discontinuation measures due to noncompliance. On the other hand, this is a well-characterized and homogeneous sample as majority of participants (96%) were APD naive.	This study will be used in the categories of long term treatment study. It will be included in analysis of clinical efficacy, extrapyramidal and other side effect, concomitant medications and discontinuation rate and reasons for; olanzapine, risperidone, haloperidol, quetiapine, ziprasidone, aripiprazole.
Gómez-	This was an open labelled trial where patients and observers were not	This study will be used in the categories of long term treatment study. It will

Citation	Study reported limitations	Reviewer Comments
Revuelta *et al.* (2020) [35]	blinded to treatments. This may have involuntarily biased the outcomes. One more limitation is that self-report and close observers was used to collect treatment adherence measures instead of APD blood levels. This could have affected the accuracy of discontinuation measures due to nonadherence.	be included in analysis of clinical efficacy, extrapyramidal and other side effect, concomitant medications and discontinuation rate and reasons for: olanzapine, risperidone, haloperidol, quetiapine, ziprasidone, aripiprazole.
Gómez-Revuelta *et al.* (2021) [31]	This study that could have involuntarily biased the outcomes are that patients and observers were not blinded to treatments Treatment compliance was either self-reported or by close observers and not from APD blood levels which could have affected the accuracy of discontinuation measures due to noncompliance. On the other hand, this is a well-characterized and homogeneous sample as majority of participants (96%) were APD naïve.	This study will be used in the categories of short term treatment study. It will be included in analysis of clinical efficacy, extrapyramidal and other side effect, concomitant medications and discontinuation rate and reasons for; aripiprazole and risperidone.
Green *et al.* (2006) [34]	This study does include the reports for the results for efficacy through neurocognitive function and brain morphology and metabolism and results for these findings along with LOCF analyses are missing in this study. The second point for consideration is majority of the participants were male (81.8%).	This study will be used in the categories of long term treatment study. It will be included in analysis of clinical efficacy, extrapyramidal and other side effect, concomitant medications and time to discontinuation for olanzapine and haloperidol. However, the results for extrapyramidal symptoms were not displayed in a table and it was only descriptive.
Grootens *et al.* (2009) [25]	Small number of patients in this study. Information about ethic approval or consent was not found in the study.	This study will be used in the categories of short term treatment study. It will be included in analysis of clinical efficacy, extrapyramidal and other side effect, concomitant medications for ziprasidone and olanzapine.
Kahn *et al.* (2008) [36]	Short wash out period was present: patients and examiners are not blinded in the study. However, no bias has been reported and this pragmatic trial reflect clinical practice which is lacking in double blinded trial. Funding was obtained from three pharmaceutical companies.	This study will be used in the categories of long term treatment study. It will be included in analysis of clinical efficacy, extrapyramidal and other side effect, discontinuation rate and time to discontinuation for haloperidol, amisulpride, olanzapine, quetiapine, ziprasidone.
Lieberman *et al.* (2003) [33]	Funding was obtained from following pharmaceutical; Novartis Pharmaceutical.	This study will be used in the categories of short and long term treatment study. It will be included in analysis of clinical efficacy, extrapyramidal and other side effect for chlorpromazine and clozapine.
Lieberman *et al.* (2003) [28]	It was an open label where patients and observers were not blinded to treatments. This may have involuntarily biased the outcomes. Funding was obtained from Lilly Research Laboratories.	This study will be used in the categories of both short and long term treatment studies. It will be included in analysis of clinical efficacy, extrapyramidal and other side effect, concomitant medications for olanzapine and haloperidol .
McEvoy *et al.*	Because EPS were minimized by reducing the APD dose as soon as possible when symptoms appeared, the scores reported for the Simpson-Angus	This study will be used in the categories of long and short term treatment studies. It will be included in analysis of clinical efficacy, extrapyramidal

Citation	Study reported limitations	Reviewer Comments
(2007) [29]	Scale, the Abnormal Involuntary Movement Scale, and the Barnes Akathisia Rating Scale represent worst-case postbaseline values. This research has received research funding or speaking fees from pharmaceuticals such as AstraZeneca, Eli Lilly.	and other side effect, concomitant medications, discontinuation rate and reasons for olanzapine, quetiapine, and risperidone. However, data for concomitant medications was only presented for in total in each medication group and data for type of concomitant medication was not presented.
Perkins *et al.* (2006) [44]	Study subjects were not epidemiological sample, therefore it may not apply to general population. clinical care differed in drug trial than routine treatment. Pill counts were used as a method of adherence measurements which is likely to be prone to errors . Also, evaluation of social support was limited and if it was more in detail, it could have affected the adherence. Measurement variability between sites could have potentially increased error. Funding was obtained from pharmaceutical such as AstraZeneca.	This study will be used in the categories of long term treatment study. It will be included in analysis of only adherence. The remaining outcomes of this study was not demonstrated in each APDs groups but rather they demonstrate results for total Health belief model. Therefore ,only information on adherence from this study could be used in analysis.
Robinson *et al.* (2015) [32]	metabolic findings may have been shown less severe since Healthy Lifestyles education program was in place. participants at entry also required to have moderate or more severe positive symptoms . in addition, the negative symptoms do not represent symptoms in absence of positive symptoms since it is only available in context of positive symptoms. Funding was obtained from pharmaceutical companies such as Bristol-Myers Squibb, Janssen/J&J, Novo Nordisk A/S, Otsuka, and Takeda.	This study will be used in the categories of short term treatment study. It will be included in analysis of clinical efficacy, extrapyramidal and other side effect, concomitant medications for aripiprazole and Risperidone.
San *et al.*, (2012) [26]	Patients and clinicians were not blind to the assigned APDs. Rates were not queried about their expectation in regard to APD efficacy. potential prescriber bias could be present due to use of hard outcome of treatment discontinuation instead of psychological scores. High rate of drop out was present and the sample size was small. The clinical assessment was performed at a fixed interval and if any discontinuation happened in between, they will still be led to the next follow up, which could be prone to bias.	This study will be used in the categories of both short and long term treatment studies. It will be included in analysis of clinical efficacy, extrapyramidal and other side effect, concomitant medications, discontinuation rate , reason and time to discontinuation for olanzapine, quetiapine, risperidone, ziprasidone, haloperidol .
Schennach-Wolff *et al.* (2011) [53]	None reported	The data of this study was not included in analysis of this meta-analysis because the outcomes were presented as remitter or non-remitter, and it was not compared across APDs.
Stauffer *et al.* (2011) [43]	None reported	The data of this study was not included in analysis of this meta-analysis because the outcomes were presented as early responders or early non responders and it was not compared across APDs.
Wang *et al.* (2017) [27]	This study was an open-label trial and bias could occur. number of patients on ziprasidone group was smaller than any other group because of non-adherence, receiving ECT, and drop-out. The efficacy was more challenging to judge, if the use of augmentation was taken. Incomplete records led to	This study will be used in the categories of short term treatment study. It will be included in analysis of clinical efficacy, extrapyramidal and other side effect, concomitant medications, discontinuation rate and reason of aripiprazole, risperidone, quetiapine, olanzapine and ziprasidone.

Citation	Study reported limitations	Reviewer Comments
	avoiding analysis of metabolic disorder and sexual dysfunction. All the reasons of discontinuation were recorded completely.	
Zipursky *et al.* (2005) [41]	large percentage of participants withdrew from the study prior completion of the 2 years period; therefore, this will cause limitation on accuracy of the weight gain estimation. Also, the result of this study is limited to only two APDs, other APDs are required to be investigated in future research.	This study will be used in the categories of long term treatment study. It will be included in analysis of weight gain for olanzapine and haloperidol.

Supplementary 4: Systematic reviews used to compare and contrast the results from this systematic review with their results.

Title and citation	Included studies	Definitions	Excluded	Comparisons
Comparative efficacy and tolerability of 32 oral antipsychotics for the acute treatment of adults with multi-episode schizophrenia: a systematic review and network meta-analysis (Huhn et al., 2019)[45].	402	Acute treatment – not defined if being due to relapse or first episode.<br>Short term: follow-up period of 3–13 weeks.<br>Mean duration of illness of 12 years	Treatment resistance, first episode, predominant negative or depressive symptoms, concomitant illnesses, relapse-prevention studies.	Placebo-controlled and head-to-head
Comparative efficacy and tolerability of 15 antipsychotic drugs in schizophrenia: a multiple-treatments meta-analysis (Leucht et al., 2013)[48].	212	Short and acute treatment defined as 6-weeks duration, when 6-week data was not available, the authors used data up to 12 weeks but not defined if being due to relapse or first episode.	Unblinded studies, negative symptoms, concomitant illness, treatment resistance, trials in patients with stable illness (relapse prevention studies).	Placebo-controlled
Antipsychotics in adults with schizophrenia: comparative effectiveness of first-generation versus second-generation medications: a systematic review and meta-analysis (Hartling et al., 2012)[46].	114	Long term defined as more than 2 years. Acute: not defined if being due to relapse or first episode.	Cohort with less than 2 years follow up.	FGAs vs. SGAs
Long-term effectiveness of oral second-generation antipsychotics in patients with schizophrenia and related disorders: a systematic review and meta-analysis of direct head-to-head comparisons (Kishimoto et al., 2019)[50].	59	Acute or maintenance/long phase. studies lasting ≥6 months included. Acute: not defined if being due to relapse or first episode.	>20% of non-schizophrenia and schizoaffective disorder. Long-acting injectable formulation long-acting antipsychotics.	Head-to-head comparisons of oral SGAs
A meta-analysis of effectiveness of real-world studies of antipsychotics in schizophrenia: Are the results consistent with the findings of randomized controlled trials? (Katona et al., 2021)[52].	18	Short duration defined as less than 24 weeks but not defined if being due to relapse or first episode.	Exclude difficult to treat or violent subjects.	Pair-wise comparisons
Efficacy and Safety of Individual Second-Generation vs First-Generation Antipsychotics in First Episode Psychosis: A Systematic Review and Meta-analysis (Zhang et al., 2013)[51].	22	First episode drug naïve patients: less than 2 weeks.<br>Long term defined as ≥1 year remission.	Maintenance studies	SGAs vs. FGAs
A Meta-analysis of the Efficacy of Second-Generation Antipsychotics (Davis et al., 2003)[47].	124	Most of the studies were short-term studies, number of long-term studies (n = 16). Acute patients but not defined if being due to relapse or first episode.	-	SGAs vs. FGAs<br>Head-to-head SGAs
Antipsychotic drugs for the acute treatment of patients with a first episode of schizophrenia: a systematic review with pairwise and network meta-analyses (Zhu, 2017)[49].	19	Acute treatment of first episode.	-	Pairwise network meta-analyses
